# PFKFB3 inhibition reprograms malignant pleural mesothelioma to nutrient stress-induced macropinocytosis and ER stress as independent binary adaptive responses

**DOI:** 10.1038/s41419-019-1916-3

**Published:** 2019-09-27

**Authors:** Sayantani Sarkar Bhattacharya, Prabhu Thirusangu, Ling Jin, Debarshi Roy, Deokbeom Jung, Yinan Xiao, Julie Staub, Bhaskar Roy, Julian R. Molina, Viji Shridhar

**Affiliations:** 1 Department of Experimental Pathology and Laboratory Medicine, Mayo Clinic, Rochester, MN USA; 2 Department of Medical Oncology, Mayo Clinic, Rochester, MN USA; 3 Division of Gastroenterology and Hepatology, Mayo Clinic, Rochester, MN USA

**Keywords:** Nutrient signalling, Mesothelioma

## Abstract

The metabolic signatures of cancer cells are often associated with elevated glycolysis. Pharmacological (PFK158 treatment) and genetic inhibition of 6-phosphofructo-2-kinase/fructose-2,6-biphosphatase 3 (PFKFB3), a critical control point in the glycolytic pathway, decreases glucose uptake, ATP production, and lactate dehydrogenase activity and arrests malignant pleural mesothelioma (MPM) cells in the G0/G1 phase to induce cell death. To overcome this nutrient stress, inhibition of PFKFB3 activity led to an escalation in endoplasmic reticulum (ER) activity and aggravated ER stress mostly by upregulating BiP and GADD153 expression and activation of the endocytic Rac1-Rab5-Rab7 pathway resulting in a unique form of cell death called “methuosis” in both the sarcomatoid (H28) and epithelioid (EMMeso) cells. Transmission electron microscopy (TEM) analysis showed the formation of nascent macropinocytotic vesicles, which rapidly coalesced to form large vacuoles with compromised lysosomal function. Both immunofluorescence microscopy and co-immunoprecipitation analyses revealed that upon PFKFB3 inhibition, two crucial biomolecules of each pathway, Rac1 and Calnexin interact with each other. Finally, PFK158 alone and in combination with carboplatin-inhibited tumorigenesis of EMMeso xenografts in vivo. Since most cancer cells exhibit an increased glycolytic rate, these results provide evidence for PFK158, in combination with standard chemotherapy, may have a potential in the treatment of MPM.

## Introduction

Malignant pleural mesothelioma (MPM), arising from the mesothelial or submesothelial cells of the serosal lining of pleura^1^, mainly due to asbestos exposure^2^, encompasses more than 80% of all mesotheliomas^3^. It has a poor prognosis with long dormancy of 30–50 years. MPM is characterized by rapid progression, high invasiveness and a very marginal impact on overall survival rate^4^. MPM has an incidence rate of 1 case per 100,000 in the U.S. with 3000 new cases per year and a median survival period of 9–12 months^5^.

Metabolic reprogramming is now documented as one of the hallmarks of cancer^6^. Increased glycolysis is one of the obligatory phenomena for neoplastic cells to thrive within the tumor micromilieu^7^. One of the critical modulators and the first committed rate-limiting factor of the glycolytic flux is 6-phosphofructo-2-kinase/fructose-2,6-biphosphatase 3 (PFKFB3)^8^. This bifunctional protein involved in both the synthesis and degradation of fructose-2,6-bisphosphate is often associated with carcinogenesis^8^, cell cycle regulation^9^, vessel sprouting^10^, and drug resistance^11^. It is overexpressed in several cancers and regulated by HIF-1α, AMPK, and MAPK^12,13^. The expression PFKFB3 is elevated in high-grade astrocytoma^14^, head and neck squamous cell carcinoma^15^, gastric^16^, thyroid, breast, colon, and in ovarian cancer^11,17^.

A major challenge for cancer cells is, how to rewire the oncogene-induced metabolic pathways to adapt in a nutrient-deficient environment^18^. Cancer cells take opportunistic approaches to acquire nutrition by engulfing extracellular macromolecules through macropinocytosis^19^, an endocytic adaptive response to nutirent stress. Macropinocytosis is associated with dysfunctional lysosomal fusion leading to extreme vacuolization of the cytoplasm called methuosis, a form of cell death^20^. Any alteration in metabolic processes also plays a critical role in cell survival due to various stresses^21^. Endoplasmic reticulum (ER) stress is another important pathway that is upregulated through glycolytic inhibition^22^.

The expression and the role of PFKFB3 are insufficiently understood in MPM. In the current study, we demonstrate for the first time that PFK158 can therapeutically target PFKFB3 to induce methuosis and ER stress. Therefore, the main objective of this study was to understand the underlying molecular mechanism of PFKFB3 inhibition and its effect in MPM cell proliferation. To the best of our knowledge this is the first report to show that by inhibiting PFKFB3 activity, we mimicked the nutrient-deficient environment in MPM which subsequently triggered macropinocytosis and ER stress with a compromised lysosomal activity. We also show PFK158-induced anti-tumor activity in MPM cells causes significant growth inhibition in preclinical models by inducing methuosis and ER stress as an independent stress response.

## Materials and methods

### Reagents

**Fig. 1:**
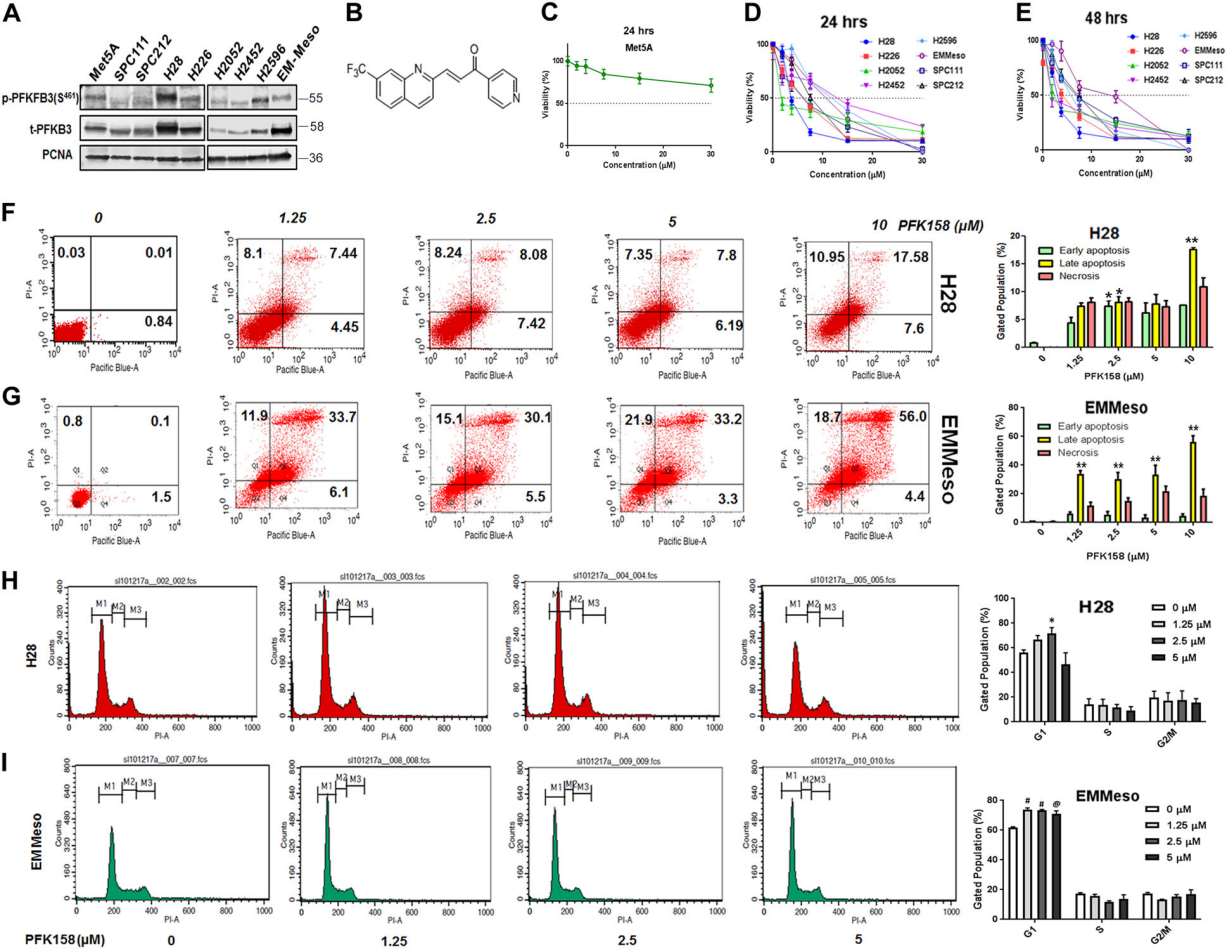
PFK158 treatment inhibits MPM cell proliferation in vitro and in vivo. **a** Western blot analysis of phospho PFKFB3 and total PFKFB3 in mesothelioma cell lines. Met5A served as normal cell control mesothelium origin. **b** Chemical structure of PFK158—a PFKFB3 inhibitor. **c** The cell viability of Met5A, an epithelial virus-transformed normal mesothelium cell line, measured by MTT assay after 48 h. PFK158 induced a decrease in pleural mesothelioma cell viability measured by MTT assay after 24 h (**d**) and 48 h (**e**). Cell viability was represented as % viability. Each value is the mean ± SD of at least three independent experiments. **f** and **g** PFK158 (0–10 μM) induced a dose-dependent increase in phosphatidylserine externalization and propidium iodide cells in H28 and EMMeso as determined by flow cytometry using annexin V-PI staining after 24 h. A representative figure, of at least three independent experiments, is shown. **h** and **i** PFK158-induced G0/G1 phase cell cycle arrest in MPM cells. A representative flow cytometry histogram of three independent experiments of cell cycle alterations after 24 h treatments in each cell line (H28 and EMMeso) is shown

PFK158 (Fig. 1b) obtained on an MTA from Gossamer Bio (San Diego, CA) was dissolved in DMSO and in 40% solution of Captisol in ddH_2_O for in vitro and in vivo studies respectively. The list of other reagents, antibodies are shown in supplementary section.

### Cell culture

Human MPM cell lines, NCI-H28, NCI-H2052, NCI-H2596 (sarcomatoid) and NCI-H226, NCI-H2452 (epithelioid) were purchased from ATCC. Among other three epitheloid cell lines EMMeso was gifted by Dr. Tobias Peikert, Mayo Clinic and SPC111, SPC212^23^ were established by Professor Rolf A. Stahel, University of Zurich, Switzerland and gifted by Dr. Jeremy Chien, University of New Mexico. All cells were authenticated by STR profiling and crosschecked with the ATCC data bank. Cells were grown in RPMI-1640 supplemented with 10% heat-inactivated fetal bovine serum (FBS) and 1% antibiotic in a humidified atmosphere at 37 °C, with 5% CO_2_. Virus transformed human mesothelium cell line Met-5A cells were also grown in RPMI-1640 with 10% FBS, epidermal growth factor (3.3 nM), hydrocortisone (400 nM), zinc-free bovine insulin (870 nM), and HEPES (20 mM)^24^.

### Cell viability assay

Cells (0.5–1 × 10^4^) were seeded in 96-well plates and exposed to PFK158 (0–30 μM) for 24 and 48 h, and the inhibitory concentrations 50% (IC_50_) values were determined by MTT assays as previously described^25^. Briefly, 3-(4,5-dimethylthiazol-2-yl)-2,5-diphenyl tetrazolium bromide (MTT, 100 μg per well) was added to fresh culture media after required drug incubation and further incubated for 3 h at 37 °C. Generated formazan crystals were dissolved in DMSO and optical density was measured at 550 nm in a multimode reader (Biotek, USA). Control cells were exposed to the highest volume of the vehicle.

### Flow cytometric assay for apoptosis

MPM cells (1 × 10^6^) were treated with PFK158 (0–10 μM) for 24 h and the externalization of phosphatidylserine was analyzed by staining the cells with Annexin V-Pacific blue and propidium iodide (5 µg/ml). Cells were acquired and analyzed by CellQuest Pro software (BD FACSCalibur) as previously described^26^. At least 50,000 cells were analyzed for each experiment with a minimum of three biological replicates.

### Cell cycle analysis via flow cytometry

MPM cells (1 × 10^6^ cells/well) were treated with PFK158 (0–5 μM) for 24 h and the cells were analyzed using a FACSCalibur flow cytometer. A minimum of 2 × 10^4^ cells per sample was evaluated, and the cell distribution percentage at each phase of the cell cycle was further calculated with CellQuestPro software^27^.

### Lactate dehydrogenase activity assay

MPM cells were cultured in the absence and presence of PFK158 (2.5–20 μM) to induce cytotoxicity and subsequently release LDH. The LDH released into the medium was transferred to a new 96-well plate and mixed with a reaction mixture according to the manufacturer’s protocol (Pierce, USA). After a 30-min room temperature incubation, reactions were stopped by adding stop solution. Absorbance at 490 and 680 nm was measured using a plate-reading spectrophotometer to determine LDH activity.

### Measurement of mitochondrial ATP production

The experiment was performed according to the manufacturer’s (BioVision Inc., USA) protocol. Briefly, 1 × 10^6^ cells (untreated and treated with PFK158, 0–20 μM) were lysed in 100 µl ATP assay buffer. 50 µl of each sample was then added to a 96-well plate. A master reaction mix was prepared (using ATP assay buffer, ATP probe, ATP converter, and developer with specified volume mentioned in the manufacturer’s protocol) and added to each well (50 µl/well) containing the ATP standard and test samples. ATP production was represented as a percentage of control by normalizing the OD values of untreated control cells.

### Cell imaging using 2-NBDG

Glucose uptake of the live MPM cells was measured using 2-NBDG as previously described^28^. Cells are plated in four-well chambered slide for overnight in complete medium. Next day, upon 60% confluency, cells were treated with PFK158 (10 µM, 0–60 min) followed by the incubation with 2-NBDG (150 µg/ml) [Cayman Chemicals, Michigan, USA] for 30 min in the glucose-free medium. Subsequently, cells were washed, mounted, and analyzed in Zeiss LSM510 fluorescence microscope. The fluorescent intensities were calculated using Image J software.

### Labeling of macropinosomes in MPM cells

MPM (parental and PFKFB3 knockdown) cells were grown in a 24-well culture plate with or without PFK158 (10 μM, 0–6 h). Media was replaced with FITC-Dextran10K incubation media (1 μg/ml) and cells were incubated at 37 °C with 5% CO_2_ cell culture incubator for 30 min. Subsequently, dextran-containing media carefully aspirated from each well followed by a gentle wash with 2 ml of ice-cold PBS for a total of five washes to ensure complete rinsing of the dye. Cells were then viewed under a fluorescence microscope.

### Intracellular Ca^2+^ measurement

MPM cell lines (H28 and EMMeso, 1 × 10^6^), treated with PFK158 (15 μM), were washed in Hanks’ balanced salt solution (HBSS) and then loaded with Fura Red (5 μM, Thermo Fisher, USA) in CaCl_2_ (1.26 mM) containing HBSS. The cells were incubated at 37 °C for 30 min in dark. All extracellular Fura red was removed by two–three times washing in the aforesaid buffer. The level of cytoplasmic Ca^2+^ within Fura red-loaded MPM cells was determined in a time-dependent manner (0–1 h) and analyzed with a FACS calibur flow cytometer (Becton Dickinson, USA). The data were analyzed with the CellQuestPro software (Becton Dickinson). Results are plotted as fluorescence changes relative to the Ca^2+^ ion chelated (2 μM EGTA) reference solution expressed as (*F*−*F*_0_)/*F*_0_, where *F* is the fluorescence intensity of ion-containing solutions and *F*_0_ is the fluorescence intensity of the reference solution.

### Immunoblot and immunoprecipitation assay

Immunoblot analysis was carried out as previously described^29^. Briefly, cells (1 × 10^6^) were treated with PFK158 (concentration-dependent and time-dependent) and 40 μg of proteins were separated in SDS–PAGE (4–12.5% gradient gel) followed by electrotransfer onto nitrocellulose membrane, blocked with 3–5% TBS–BSA, probed overnight with primary antibodies (Supplementary information) at 4 °C and washed with 0.1% Tween-20-containing TBS. Immunocomplexes were identified with fluorophore-conjugated secondary antibodies (LI-COR). The membrane was washed and target proteins were identified by the LI-COR OdysseyFc Imaging System (Nebraska, USA).

For detection of the protein complex, the cell lysates containing 400 μg of protein were incubated with the anti-Rac1 antibody (1:100) overnight at 4 °C, and then 10 μl of 50% protein A-agarose beads were added and thoroughly mixed at 4 °C for 6 h. The immunoprecipitates were washed thrice with chilled PBS, collected and precipitated beads were loaded into the sample buffer, subjected to electrophoresis on 4–12.5% SDS–PAGE and blotted using an anti-Rab7 or anti-Calnexin or anti-Rac1 antibody.

### Reverse phase protein array (RPPA)

In order to identify additional novel or known markers modulated by PFK158 in MPM, we performed RPPA at MD Anderson Cancer Center, Houston, TX. Briefly, 0.3–0.5 × 10^6^ cells/2 ml MPM cells were seeded in six‐well plate for overnight followed by the treatment with IC_50_ of the PFK158 at 24 h for each cell line in triplicate. Subsequently, cells are washed in PBS and lysed in lysis buffer provided by MD Anderson Cancer Center. The cell lysate was centrifuged in a microcentrifuge at 14,000 rpm (maximum speed) for 10 min at 4 °C. Cellular protein concentration was determined by the Bradford reaction and at least 40 μl (concentration 1.5 μg/μl) protein was used for each sample. Three parts of cell lysate were mixed with one part of the sample buffer (MD Anderson Cancer Center), boiled for 5 min, and stored at −80 °C until sample submission.

### Generation of PFKFB3 downregulated stable clones

PFKFB3 downregulation was performed in H28 and EMMeso cells with ShPFKFB3 [Sh55: CGGGTGCATGATTGTGCTTAA (targeting 3′UTR), Sh59: CCACCAATACTACTAGAGAGA (targeting 5′UTR)] using standard transfection protocol and reagents.

### Immunofluorescence (IFC) assay

MPM cells, untreated and treated with PFK158 or PFKFB3 downregulated cells were grown in four-well chambered slide for the desired time and fixed with 4% paraformaldehyde at 4 °C for 10 min. Cells were then washed followed by blocking with 3% PBS–BSA at 37 °C for 1 h. Subsequently, cells were probed with the primary antibody in 3% PBS–BSA (1:200 dilution) at 4 °C for overnight. Later incubated with secondary antibody in 3% PBS–BSA (1:200 dilution) at 37 °C for 1 h. Immunostained cells were mounted with mounting medium containing DAPI (1.5 μg/ml) (Vectashield, USA) and visualized by using Zeiss-LSM 510 confocal microscope. Quantification of the fluorescence was measured using Image J software.

### Tumor xenograft study

Twenty-four female athymic homozygous nude mice (nu/nu, 4–8 weeks old mice) were obtained from Jackson Laboratories, USA. After 1-week acclimatization, the mice were randomized in four groups (*n* = 6) and EMMeso cells (5 × 10^6^ in 200 μl of PBS) were injected subcutaneously (subQ) on the right flank of the fore-limb. Five days following the tumor cell inoculation, the mice were treated with: (A) 40% Captisol for control group, (B) 30 mg/kg of PFK158 twice in a week for 2 weeks, (C) 50 mg/kg of CBP once in a week, and (D) combination of CBP (50 mg/kg once a week) and PFK158 (30 mg/kg twice in a week). After a 2-week treatment, mice were kept under observation for 5 days and all mice were sacrificed on day 21, considering the first dose of treatment as day 1. Mice body weight and tumor volume were measured throughout the study. Finally, after sacrifice, body weight, tumor weight, and tumor volume were determined. Tumors were preserved either in formalin or −80 °C or Trump’s fixative. The whole animal experiment was performed complying with the guidelines of the Institutional Animal Care and Use Committee (IACUC) at the Mayo Foundation, in accordance with approved protocols.

### Immunohistochemistry assay

For immunohistological staining, cryo-preserved tumors were fixed, embedded in paraffin, and microtome-sectioned. Tissue sections were stained with anti-ki67, anti-Rac1, and anti-BiP antibodies.

### Transmission electron microscopy (TEM) analysis

MPM cells (untreated and treated with PFK158 or PFKFB3 downregulated cells) and tumors from xenograft study were fixed in Trump’s fixative and submitted to the Microscopy and Cell Analysis Core facility in Mayo Clinic, MN for further processing. Images were taken in JEM-1400 transmission electron microscope (Jeol, USA).

### Statistical analyses

All results were expressed as mean ± standard deviation. Data were obtained from at least three separate experiments. All statistical analyses were performed using the GraphPad Prism 7.05 software (San Diego, CA). Data were analyzed using either non-linear regression or *t*-test or one-way ANOVA as appropriate. A level of *P* < 0.05 denoted as *<0.002 as **<0.0002 as *** or ^**@**^ and <0.0001 as **** or ^**#**^ considered as statistically significant unless otherwise mentioned. *P* > 0.05 considered as non-significant (ns).

## Results

### PFK158 treatment inhibits MPM cell proliferation in vitro

We recently reported that activated PFKFB3 levels are high in gynecological cancers^11^. To understand the role of PFKFB3 in MPM, we first determined the basal and activated form of PFKFB3 (p-PFKFB3-S^461^) expression in all MPM cell lines (Fig.1a). Irrespective of their histology or mutational status, PFK158 (Fig.1b) treatment inhibited MPM cell viability [including the control virus-transformed mesothelium cell line, Met5A that showed negligible effect, even at a 30 µM dose (Figs.1c and S1)] in a time-dependent and dose-dependent manner (Fig. 1d, e). The IC_50_ values ranged from 3 to 12 μM at 24 h of treatment. DMSO (0.05%) served as the vehicle control and exhibited a minimal adverse effect.

### PFK158 treatment induces apoptosis by arresting MPM cells at G0/G1 phase of cell cycle

Flow cytometry analysis using Annexin V and PI labeling showed that PFK158 treatment for 24 h of H28 (Fig. 1f), EMMeso (Fig. 1g), and H2452 (Fig. S2A) cells resulted in an increase in early (~20%) and late (~35%) apoptosis in a dose-dependent manner with very low necrotic cells (~2%). Under the same condition, western blot analysis showed a significant increase in the level of cleaved poly [ADP-ribose] polymerase (PARP) in a dose-dependent manner in MPM cell lines (Fig. S2B).

Notably, we observed that the metabolic activity and the doubling time of EMMeso cells are shorter compared to H28, that displayed delayed cell doubling time (~36 h), which could account for the differences in their IC50 values.

To determine whether PFK158-induced growth inhibition was associated with cell cycle arrest in a particular phase, we performed flow cytometry following PFK158 (0–5 µM) treatment and representative data of H28 and EMMeso cells are shown in Fig. 1h, i. In untreated H28 cells, 67.89%, 10.77%, and 15.79% cells were in the G0/G1, S, and G2/M phases, respectively. PFK158 treatment resulted in a significant accumulation of cells in the G0/G1 phase with a striking reduction of cells in the G2/M phase. The peak of arrested cells in the G0/G1 phase was observed at the 2.5 µM dose up to 73.16% following 24-h treatment. Similar results were seen in EMMeso cells with 66.25%, 15.82%, and 17.05% of cells, which were in the G1, S, and in the G2/M phase, respectively. Similar to H28 PFK158 treatment arrested cells in the G0/G1 phase (73.75%) and a remarkable reduction of cells in the S (11.48%) and G2/M phases (14.25%) at 2.5 µM. Collectively, these results indicate that PFK158 induces apoptotic cell death by arresting them in G0/G1 of the cell cycle.

### PFK158 treatment leads to a decrease in glycolytic rate and inhibits p-PFKFB3 levels

**Fig. 2:**
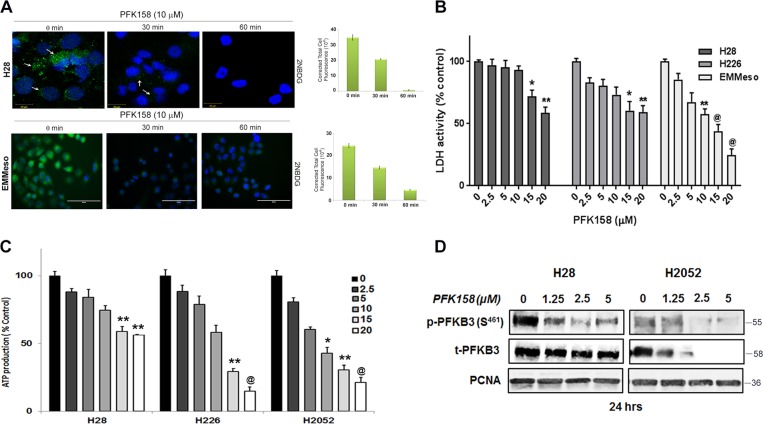
PFK158 treatment prevents glycolysis in MPM by inhibiting active PFKFB3 MPM cells evidently increased glucose uptake, LDH activity, intracellular ATP, and phospho-PFKFB3 (S^461^) level, which is inhibited by PFK158 treatment. **a** Fluorescence images of glucose uptake using 2-NBDG in H28 and EMMeso cells. LDH activity (**b**) and intracellular ATP (**c**) were measured in H28, H226, H2052, and EMMeso cells in the presence and absence of PFK158 (0–20 μM), where *P < 0.05, **P < 0.05 and ^**@**^*P* < 0.0001. All experiments were conducted in eight replicates and for at least two times. Data are shown as mean ± SD for eight replicates per cell line. **d** A dose-dependent decrease in the level of active PFKFB3 [phospho-PFKFB3 (S^461^)] after PFK158 treatment

To evaluate if PFK158 inhibits glycolytic rate, we determined glucose uptake, lactate dehydrogenase activity, and intracellular ATP level in MPM cell lines in the presence and absence of PFK158. Results showed that a concentration-dependent decrease in glucose uptake in H28, EMMeso, and SPC111 as determined using 2NBDG, a fluorescent glucose analog (Figs. 2a and S2C), respectively. Consistent with a decrease in glucose uptake, PFK158 showed a reduction in lactate dehydrogenase activity (Fig. 2b) and intracellular ATP levels (Fig. 2c) a dose-dependent decrease in p-PFKFB3 (S^461^) by immunoblot analysis (Fig. 2d) suggesting PFK158 inhibits the glycolytic rate in MPM cells.

However, PFK158 treatment neither triggered reactive oxygen species (ROS) or promoted autophagy in MPM cells (Fig. S3A, B). Also PFKFB3 knockdown cells showed negative β-galactosidase staining, suggesting that PFK158-mediated or PFKFB3 knockdown-mediated glycolysis inhibition is dispensable for senescence in MPM (data not shown).

### Pharmacological and genetic inhibition of PFKFB3 leads to increased macropinocytosis

**Fig. 3:**
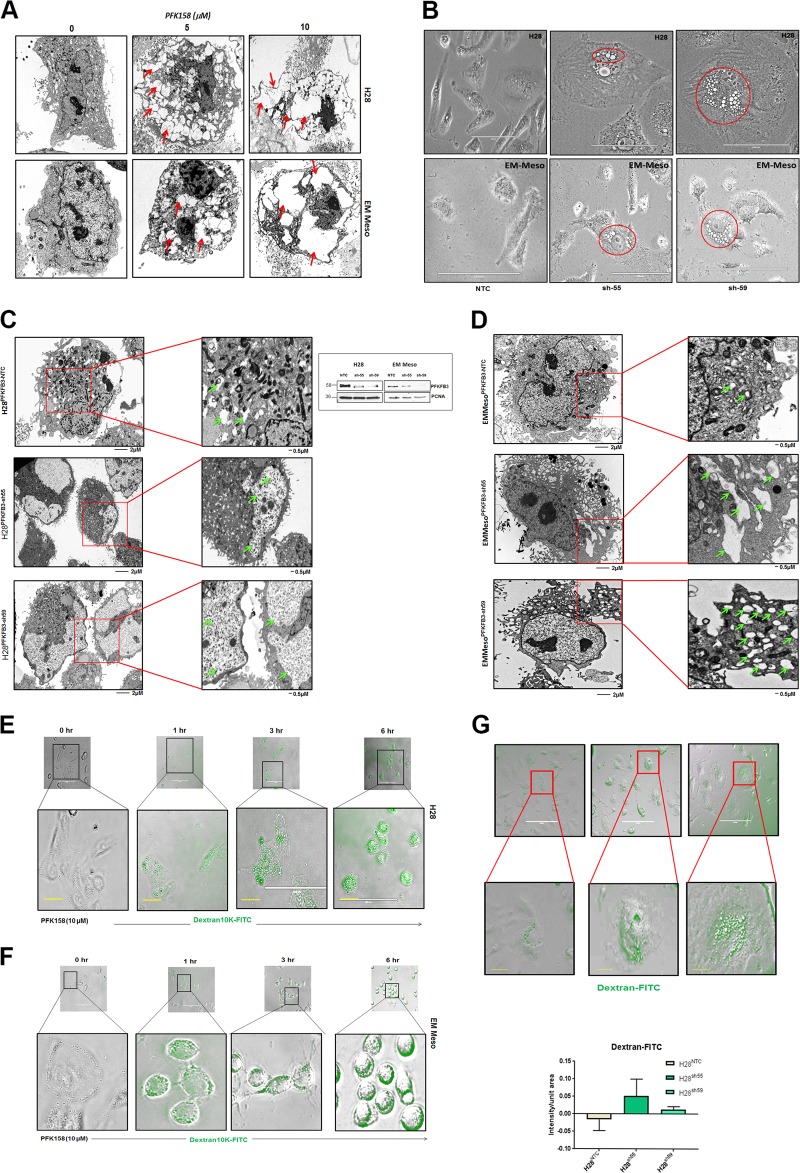
Inhibition of PFKFB3 leads to increased macropinocytosis **a** Transmission electron microscopy (TEM) revealed a dose-dependent increase in vacuolization in MPM cells after PFK158 treatment (0–10 μM) in H28 and EMMeso cells. **b** Phase contrast microscopic and **c**, **d** TEM pictures of increased vacuoles in PFKFB3 knockdown clones (sh55 and sh59) in comparison to non-targeted control (NTC). **e** and **f** Augmented macropinocytosis visualized by Dextran-10K uptake after PFK158 treatment both in H28 and Meso. **g** PFKFB3 knockdown H28 clones also demonstrated extensive macropinocytosis by Dextran-10K uptake

Metabolic reprogramming is increasingly recognized as one of the hallmarks of cancer^6^. It has been reported that macropinocytosis may occur as a metabolic adaptive response to nutrient stress^18^, The TEM analysis showed inceasing vacuole formation both in pharmacologically inhibited (Fig. 3a) and genetically knockdown (KD) (Fig. 3c, d) MPM cells. The PFKFB3KD cells formed much bigger vacuoles which could be scoped in a phase-contrast microscope at ×40 magnification (Fig. 3b). Macropinosomes can be visualized based on the ability of cells to internalize extracellular fluorescently labeled high molecular weight dextran (FITC-dextran 10K). Our data showed a time-dependent increase in FITC-dextran uptake both in PFK158-treated H28 and EMMeso cells (Fig. 3e, f), respectively. In parallel, genetic knockdown of PFKFB3 in H28 (PFKFB3^KD^H28) cells also displayed appreciably higher levels of dextran uptake compared to non-targeted control transduced cells (Fig. 3g). Western blot analysis shows efficient knockdown of PFKFB3 using two different shRNAs in both H28 and EMMeso cells (inset of Fig. 3c, d).

### PFKFB3 inhibition triggers molecular mediators of macropinocytosis

**Fig. 4:**
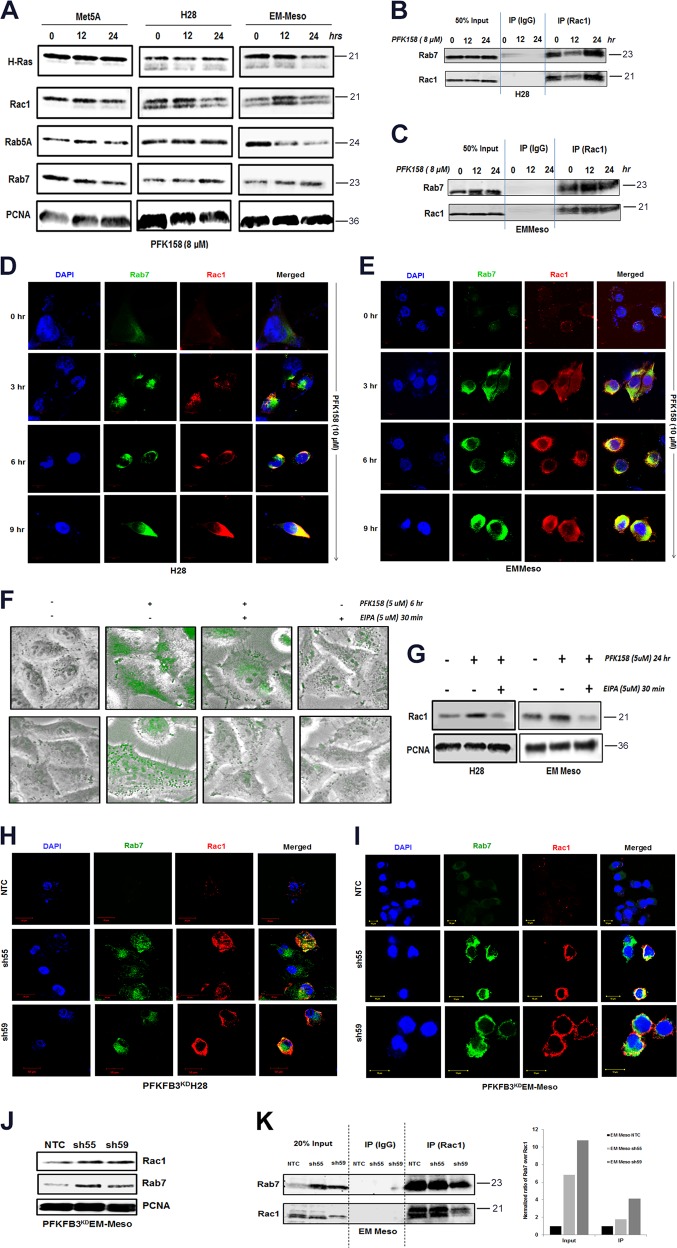
PFKFB3 inhibition triggers molecular mediators of the endocytic pathway **a** Met5A, H28, and EMMeso cells were treated with PFK158 (8 μM) for 0, 12, and 24 h and cell lysates were evaluated with immunoblot analysis using anti-H-Ras, anti-Rac1, anti-Rab5A, anti-Rab7, and anti-PCNA antibodies. Increase in Rac–Rab7 interaction after PFK158 treatment as evident by immunoprecipitation with anti-Rac1 and subsequent immunoblotting with anti-Rac1 and anti-Rab7 separately both in H28 (**b**) and EMMeso (**c**)**. d** and **e** Confocal microscopic images revealed increased Rac1–Rab7 interaction after PFK158 treatment (0–9 h). **f** NHE inhibitor EIPA-inhibited macropinocytosis in MPM cells visualized by Dextran-10K uptake. **g** Immunoblot analysis of Rac1 after EIPA treatment. Confocal images of PFKFB3 knockdown clones both in H28 (**h**) and in EMMeso (**i**) showed increased interaction of Rac1 and Rab7. **j** Western blot showed increased Rac1 and Rab7 in PFKFB3^KD^EMMeso cells. **k** Co-immunoprecipitation assay with anti-Rac1 following immunoblot with anti-Rab7 in PFKFB3 knockdown EMMeso clones (sh55 and sh59) also revealed increased interaction with these two proteins

To further elucidate the molecular players of macropinocytosis in PFK158-treated MPM cells, we determined the expression of Rac1 and Rab7 in the endocytic pathway in PFK158-treated Met5A, H28, and EMMeso cells. Western blot analysis showed a time-dependent upregulation of Rac1 and Rab7 in PFK158-treated H28 and EMMeso cells (Fig. 4a), with no significant change in H-Ras and Rab5A levels. There were minimal changes in the expression of any of these markers in Met5A-treated cells.

Previous studies have reported that Rac1–Rab7 interaction may mediate late endosomal transport because Rac1 is involved in the remodeling of the cytoskeleton that leads to macropinocytosis through its effector Rab7. To understand if PFK158 treatment triggers late endosome formation, we performed immunoprecipitation of Rac1 following PFK158 treatment (8 µM, 12 and 24 h) and immunoblotted with anti-Rab7 and anti-Rac1 antibodies in both H28 and EMMeso cells. The results showed a moderate increase in Rac1–Rab7 interaction at these time points (Fig. 4b, c). However, when this data was further validated by immunostaining of Rac1 and Rab7 in earlier time points (0–9 h) and their colocalization was visualized in confocal imaging (Fig. 4d, e) significant increase in the intensity of Rac1 and Rab7 as shown in Fig. S4A–D.

Moreover, PFK158-mediated macropinocytosis was confirmed by pre-treating MPM cells with EIPA, a canonical Na^+^/H^+^ exchange (NHE) inhibitor that inhibits macropinocytosis. EIPA efficiently inhibited PFK158-induced fluid phase dextran uptake and Rac1 upregulation in both H28 and EMMeso as shown in Fig. 4f, g, respectively. However, the normal mesothelial, Met5A cell line, did not show any substantial activation of the endocytic pathway (Fig. S5A, B). In parallel, PFKFB3^KD^H28 and PFKFB3^KD^EMMeso cells also displayed substantially higher levels of Rac1 and Rab7. Consistent with these results, using shNTC and shPFKFB3 cells in both H28 and EMMeso cells, confocal imaging (Fig. 4h, i) showed knockdown of PFKFB3 resulted in increased interaction of Rac1 with Rab7. Western blot (Fig. 4j) and immunoprecipitation assay (Fig. 4k) in PFKFB3^KD^EMMeso cells further confirmed upregulated Rac1, Rab7, and the increased interaction of Rac1 with Rab7, respectively.

### Glycolytic inhibition by PFK158 is accompanied by the changes in ER-associated proteins

**Fig. 5:**
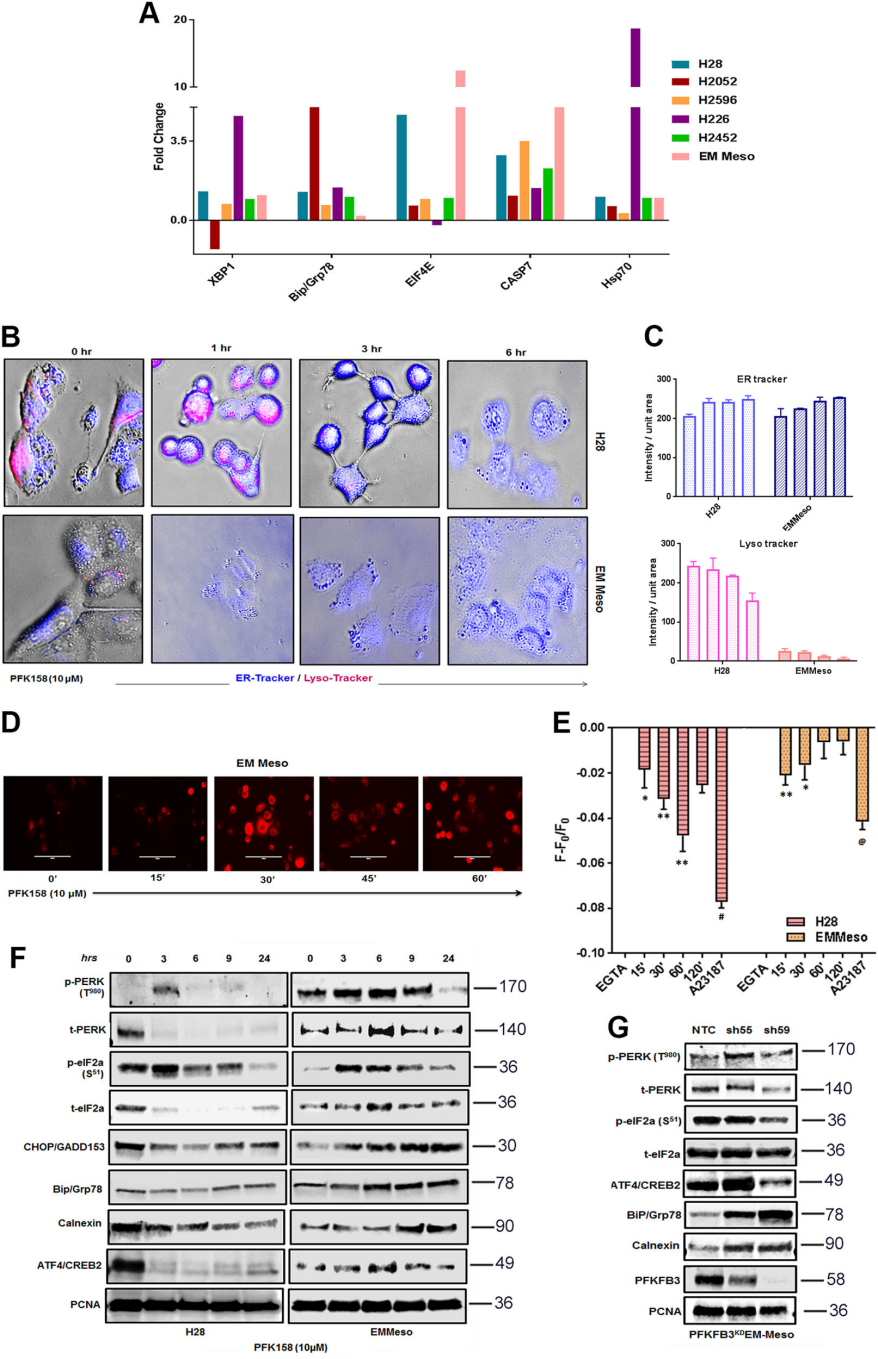
PFK158 initiates unfolded protein response in MPM cells **a** Fold changes in most of the UPR-related proteins after treatment with IC_50_ of the PFK158 of each cell line at 24 h, followed by large-scale analyses of changes in protein level and their modification using reverse-phase protein arrays (RPPA) as described in the “Material and methods” section. One-way ANOVA was used to analyze the data. **b** ER and lysosomal activity were visualized by confocal microscopy stained with ER-tracker blue/white DPX and lysotracker red in H28 and EMMeso cell treated with PFK158 (10 μM) for different time point (0–6 h). **c** Graphical representation of the increase in ER-tracker blue and decrease in lysotracker red. MPM cells were loaded with Fura Red to stain intracellular Ca^2+^ pool and either observed under a fluorescent microscope **d** or analyzed by FACS **e**. Increase in ER stress marker proteins after pharmacological inhibition **f** and genetic knockdown **g** of PFKFB3 as demonstrated by Western blot

Even though PFK158 was developed as a specific inhibitor of PFKFB3, recent data indicate that it may have off-target effects^11^. To determine the mechanism of action of PFK158 in MPM cells, we performed a large-scale analysis of changes in protein level and their modification using reverse-phase protein arrays (RPPA) as described in the “Materials and methods” section to verify the intracellular pathways significantly deregulated by PFK158 treatment in six MPM cell lines. Our analysis revealed that the proteins involved in the endoplasmic reticulum stress, viz. XBP-1, Bip-Grp78, phospho e-IF4E, Hsp27, Caspase 7, and Hsp70 were upregulated in the majority of the cell lines following PFK158 treatment (Fig. 5a). These data suggest that PFK158 may induce ER stress in MPM cells. To validate the results from the RPPA analysis, we monitored ER activity using the ER-Tracker Blue-White DPX, a cell-permeable dye that selectively labels the ER. 10 µM PFK158 treatment led to a significant increase in ER activity as measured by the intensity of ER-Tracker Blue-White DPX in both H28 and EMMeso cells (Figs. 5b, c and S6) in a time-dependent manner. The quantitation of the signal intensity is shown as a bar graph. However, under the same experimental conditions, labeling for tracking the acidic compartment, the lysosomes with Lysotracker Red showed a decrease in the intensity of staining (Fig. 5b, c). Collectively, these data indicate that while PFK158 induces ER stress, it seems to suppress lysosomal activity.

### A time-dependent upsurge of intracellular Ca^2+^ in MPM cells

As inhibition of glycolysis can lead to calcium overload^30,31^, we further measured the intracellular calcium pool in PFK158-treated cells. The maximum increase in intracellular Ca^2+^ pool in H28 and in EMMeso was in 60 and 15 min after treatment, respectively (Fig. 5d, e). EGTA (10 mM) and Ca^2+^ ionophore (2 μM) served as negative and positive controls, where it showed the lowest and the highest mean fluorescence intensity (MFI), respectively. These results confirmed that PFK158 may induce the release of Ca^2+^ from the ER to activate ER stress in these cells.

### PFKFB3 expression inversely correlates with ER stress response in MPM

To further elucidate the protein players of the ER stress pathway, we checked an array of ER stress markers by Western blot analysis. PFK158 treatment lead to the upregulation of activated PERK (T^980^) and eIF2a (S^51^), as well as upregulation of BiP as early as 3 h and GADD153 by 9 h of treatment. However, ER chaperone calnexin was upregulated in EMMeso significantly (Fig. 5f). Similar results were observed also in PFKFB3 knockdown in EMMeso cells. The ER stress was found to be more prominent in this cell line (Fig. 5g). However, in the sarcomatoid H28^PFKFB3KD^ cells it did not show significant upregulation of ER stress proteins (data not shown).

### Activation of ER stress and macropinocytosis are two independent adaptive responses after glycolytic stress

**Fig. 6:**
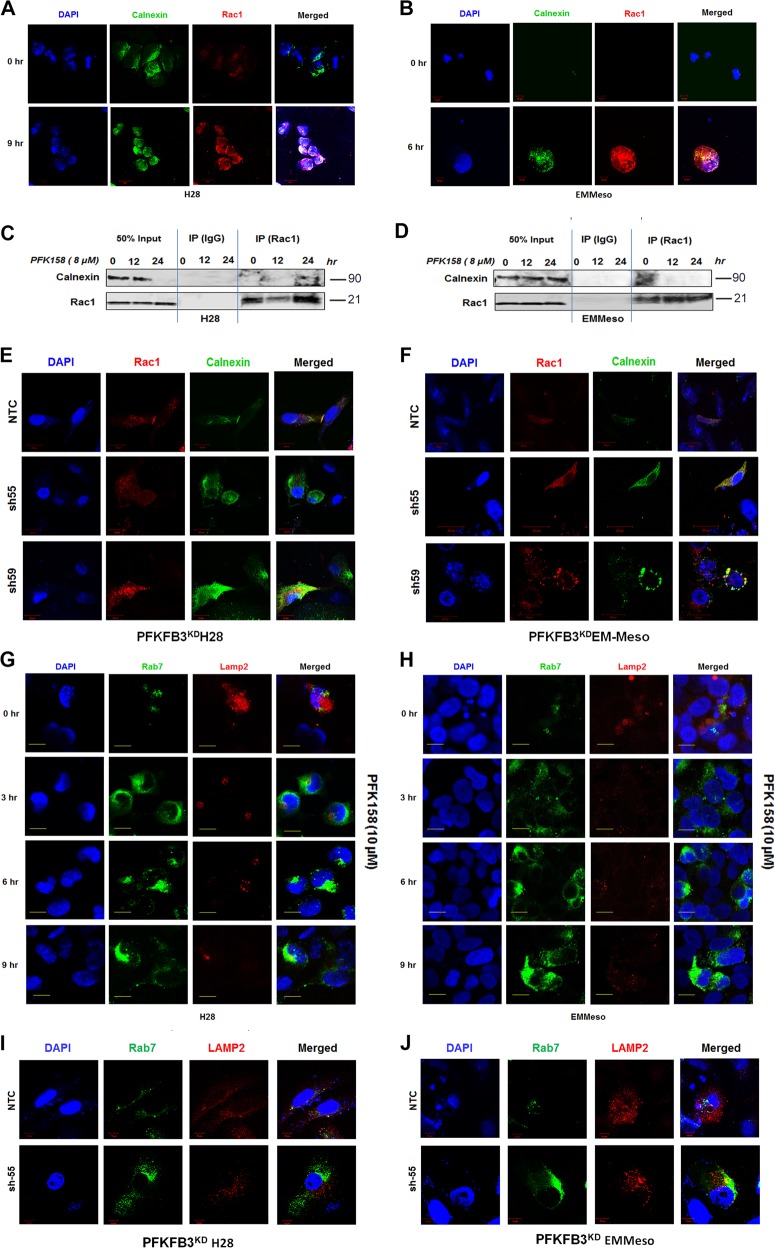
ER stress and macropinocytosis with impaired lysosome are unrelated responses after glycolytic stress Confocal microscopic images demonstrated least interaction of Calnexin (ER marker) and Rac1 (macropinocytosis) after PFK158 treatment (10 μM), both in H28 **a** and EMMeso **b** cell lines in early time points. **c** and **d** This interaction further evaluated by co-immunoprecipitation assay with anti-Rac1 followed by immunoblotting with anti-Calnexin in these cell lines. **e** and **f** Confocal microscopic images for Colocalization of Rac1 and Calnexin in PFKFB3 knockdown H28 and EMMeso clones. Costaining of late endosome marker, Rab7 and lysosome marker Lamp2 showed impaired interaction after PFKFB3 inhibition both either by PFK158 treatment **g**, **h** or by genetic knockdown **i**, **j**

Till now we have established that the inhibition of PFKFB3 elicits macropinocytosis and ER stress as probably two adaptive responses towards glycolytic stress. To further clarify that the inhibition of glycolysis and these two adaptive responses were linked together or independent of each other, we performed several colocalization studies. Co-staining of engulfed high molecular weight Dextran (10K) with ER marker calnexin clearly showed no co-localization of these markers both in H28 and EMMeso (Fig. S7A, B), though these were upregulated after PFK158 treatment in a time-dependent manner. We subsequently assessed whether Rac1, involved in the early stages of macropinosome formation and calnexin interact with each other. Both the confocal images (Fig. 6a, b) of the Rac1–Calnexin colocalization, and time-dependent (Fig. 6c, d) and concentration-dependent (Fig. S7C, D) immunoprecipitation assay clearly demonstrated that they were not interacting with each other after PFK158 treatment. In addition, PFKFB3 knockdown H28^PFKFB3KD^ and EMMeso^PFKFB3KD^ also revealed minimal to no colocalization of these two proteins (Fig. 6e, f). However, EHT1864, a Rac1 inhibitor, could not rescue MPM cells from ER stress, in term of reduction of Bip/Grp78 downregulation (data not shown), suggesting that the activation of macropinocytosis and ER stress are a result of two independent PFKFB3-modulated adaptive responses.

### Impaired macropinosome–lysosome fusion leads to methuosis in MPM upon PFKFB3 inhibition

Our data show that PFK158 mediated escalation in intracellular vesicles, which coalesced to form huge vacuoles (Fig. 3), as well as lysosomal inactivity occur in parallel (Fig. 5b, c). These two observations led us to hypothesize that PFK158 may block lysosomal processing of these vesicles. To check whether these vesicles are apportioned to the lysosome or not, we co-immunostained the treated cells with Rac1 and Lamp2 as specific markers of their respective cellular compartments. The data showed that PFK158 treatment resulted in a gradual decrease of Lamp2 with an increase of Rab7 in a time-dependent manner (Fig. 6g, h). A similar phenomenon was also seen in PFKFB3 knockdown MPM cells (Fig. 6i, j). These data confirmed that PFKFB3 inhibition leads to an impaired macropinosome–lysosome fusion, which probably causes the fusion of the vacuoles to displace the cytoplasm leading to cell death. This suggests that compromised vesicle recycling upon PFKFB3 inhibition both pharmacologically or genetically may trigger a nonapoptotic cell death “methuosis” in MPM.

### Pharmacological and genetic inhibition of PFKFB3 leads to microtubule disassembly

As methuosis often is associated with deregulation of cytoskeletal proteins, we, subsequently analyzed tubulin remodeling after PFKFB3 inhibition. Confocal imaging confirmed α-tubulin disassembly after PFK158 treatment and genetic knockdown of PFKFB3 both in H28 and EMMeso cells (Fig. S8A, B).

### PFK158 alone is a potent suppressor of in vivo MPM tumor growth

**Fig. 7:**
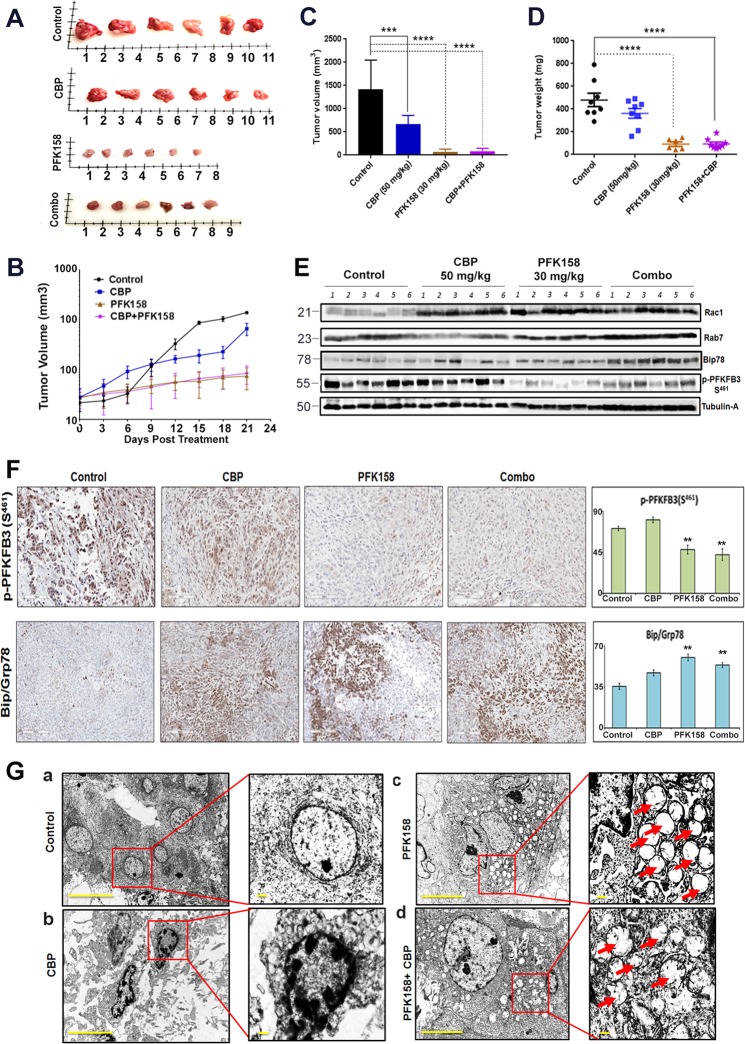
PFK158 alone is a potent suppressor of in vivo MPM tumor growth **a** The reduction in tumor size as reflected in the image of six representative MPM xenograft tumors, excised after 21 days of vehicle, Carboplatin, PFK158, and combination treatment. The decrease in MPM tumor growth (by time) **b**, tumor volume **c**, and tumor weight **d** after 21 days of respective treatments. **e** Immunoblot analysis against Rac1, Rab7, Bip, and p-PFKFB3 (S^461^) in MPM xenograft tumors. **f** Representative images of immunohistochemical staining of phospho-PFKFB3 (S^461^) and Bip/Grp78 performed in MPM xenograft tumor tissue after individual and combinatorial treatments. **g** **(a–d**) TEM images of xenograft tumor revealed increased vacuolization in PFK158-treated mice either singly or in combination with CBP

To further investigate the activity of PFK158 alone and/or in combination with carboplatin (CBP) in an MPM xenograft nude mouse model, we implanted EMMeso cells subcutaneously in nude mice (Fig. S9A). A noticeable reduction in tumor burden (Fig. S9B and Fig. 7a), tumor growth (Fig. 7b), tumor volume (Fig. 7c), and tumor weight (Fig. 7d) were observed in both in PFK158 alone and in combination treatment. We also observed that the tumor burden in PFK158-treated mice was significantly (*P* < 0.0002) less than the one in the vehicle-treated controls. However, our data also showed that the PFK158 alone was profoundly effective as it was in combination with CBP treatment in reducing pleural mesothelioma progression compared to CBP single treatment group. No significant decrease in body weight was observed in any of the groups (Fig. S9C).

**Fig. 8:**
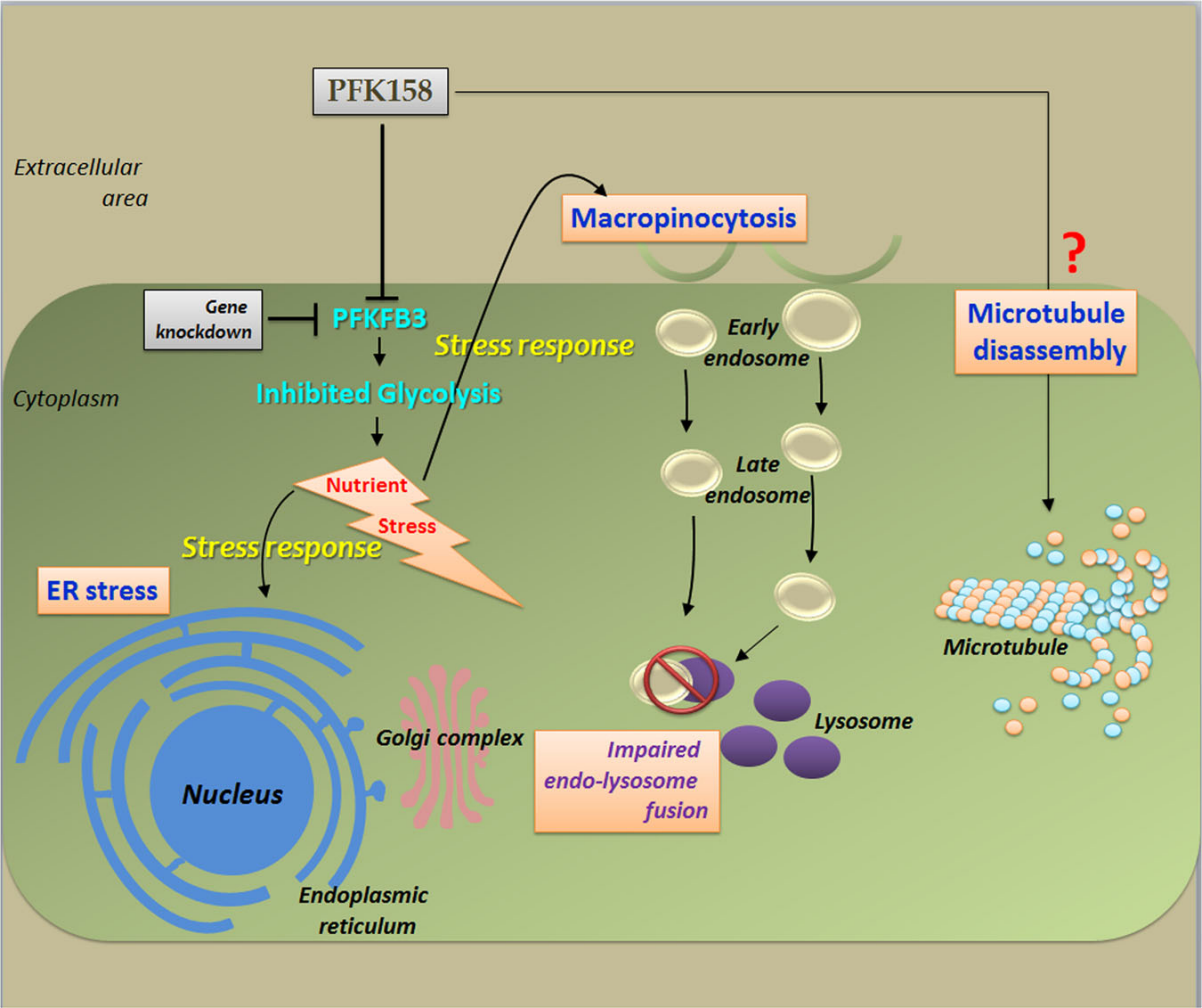
Two independent adaptive responses displayed after interfering glycolysis via PFKFB3 inhibition. Firstly macropinocytosis, by which cancer cells acquire the raw materials for anabolism with a subsequent unachieved goal due to impairment of endo-lysosomal trafficking; secondly ER stress, to perturb the manufacturing and packaging system of the cell

Furthermore, expression of key modulatory proteins from xenograft tumor lysate also showed consistency with the in vitro data. Western blot analysis also revealed an increase in Rac1, Rab7, and Bip/Grp78 expression. However, there was a significant decrease in expression of p-PFKFB3(S^461^), a PFK158-targeted protein (Fig. 7e). Western blot data were further substantiated by IHC staining of xenograft tumors with p-PFKFB3(S^461^) and Bip/Grp78 (Fig. 7f). Additionally, TEM of xenograft tumors also revealed profound methuotic vacuoles both in PFK158-alone and in combination treatment [Fig. 7g(a–d)]. Together the in vivo data revealed that PFK158-mediated inhibition of tumorigenesis occurs through methuosis and ER stress to reduce the tumor burden.

## Discussion

Principally, metabolism is an assembly of self-supporting chemical conversions to provide energy within the cells. Constitutive and persistent upregulation of glycolysis is one of the major metabolic pathways during the progression of in situ neoplasia to invasive cancers^32^. Suppressing glycolysis by selective inhibition of PFKFB3 isozyme is considered to be a promising therapeutic target due to its strong allosteric activity towards PFK1, which stimulates glycolysis and often overexpressed in most of the cancers. To the best of our knowledge, this is the first study to establish that inhibition of PFKFB3 with a small molecule antagonist, PFK158; can introduce glycolytic assault which simultaneously triggers ER stress and methuosis and eventually suppress MPM cell growth both in vitro and in vivo (Fig. 8).

Glucose is one of the major nutrients which offers energy and/or building blocks for neoplastic proliferation^33^. We observed that PFK158 targets the glycolytic pathway, which results in glucose depletion and eventually nutrient deficiency in MPM cells. Highly aggressive cancers, viz. pancreatic cancer, employ macropinocytosis to overcome the nutrient scarcity by scavenging supplementary nutrients, predominantly proteins and lipids^34^. This endocytic pathway mainly coupled with subsequent lysosomal processing as an important substrate-acquisition route. Conversely, we have seen that though PFKFB3 inhibition triggered macropinocytosis as an adaptive reaction after glycolysis arrest, yet the consecutive lysosomal processing was hindered leading to an enrichment of coalescing macropinosome without resolving nutrient-deficiency issue.

Moreover, glucose metabolism is the main source of protons which is a pre-requisite factor for the mitochondrial function^35^. Compromised glycolysis leaves cells with proton scarcity despite the fact that the mitochondrial electron transfer chain remains consuming protons to produce energy. It promotes a compensatory lysosomal proton efflux and subsequently increased in lysosomal pH. This lysosomal alkalization compromises its enzymatic inactivity. A similar situation also developed after PFK158 treatment and/or inhibition of PFKFB3 activity. Though MPM cells initially tried to surpass the nutrient deficit by activating Rac-Rab endocytic pathway, still lysosomal inactivation hindered the processing of exogenous nutrients and consequently making them more susceptible to cell death. Thus PFKFB3 inhibition can trigger methuosis along with apoptosis in MPM. Remarkably, a very recent study revealed that chalcones, known to be methuosis inducers, also can adversely interfere with glucose uptake^36^. We are the first to show a metabolic connection between glycolysis with elevated macropinocytosis and impaired lysosomal function to induce methuosis and reveal a crucial role of glucose metabolism in maintaining nutrient and energy homeostasis to sustain cell survival.

Furthermore, disturbance in glucose homeostasis enforces a severe threat countered by activation of stress responses to abate the damage and restore the energy balance^37^. The ER is one of the important organelles that play an essential role in maintaining the protein and Ca^2+^ homeostasis^38^. Inhibition of glycolysis^39,40^ or induction of ER stress^21^ often found to be associated with ER stress and glycolysis inhibition correspondingly. We observed PFK158-mediated glycolysis assaults also can trigger alterations in intracellular Ca^2+^ homeostasis and ER stress in MPM. The altered glycolytic pathway in treated cells can reinforce the dysfunction of prime activities of ER. Recent evidence also supports the inverse association of PFKFB3 and ER stress^41^. Considering these, we theorize that glucose deprivation via PFKFB3 inhibition resulting in energy depletion which activated unfolded protein response (UPR), as one of the major stress responses; to restore proteostasis. However, another intracellular stress response, ROS along with autophagy, found to be decreased in MPM after PFK158 treatment and these are also consistent with the results observed in myeloproliferative neoplasm and T cells from rheumatoid arthritis patients^42–45^.

Likewise, some methuosis-inducing chalcones which also can disrupt microtubule^46^, PFK158, a glycolytic inhibitor, can trigger methuosis and also found to disrupt the microtubule assembly in MPM.

Ultimately, in this study, we define two adaptive responses displayed after interfering glycolysis, one of the key metabolic pathways. Firstly macropinocytosis, by which cancer cells acquire the raw materials for anabolism with a subsequent unachieved goal due to impairment of endo-lysosomal trafficking; secondly ER stress, to perturb the manufacturing and packaging system of the cell. However, though some crucial open questions yet to be addressed before the safe and successful employment of apical metabolic inhibitors in the clinic, yet this study opens some avenues to target MPM, by metabolic inhibitors highlighting some of the targetable critical proteins in each pathway. In summary, the recent study provides strong support that nutrient limitation along with interventions that disrupt nutrient acquisition by critical metabolic adaptation is a potential therapeutic strategy to efficiently and safely starve MPM cells to death and can make a platform for new chemotherapeutic agents that exploit the nutrient dependencies of cancers.

## Supplementary information

Supplemental Figure S1

Supplemental Figure S2

Supplemental Figure S3

Supplemental Figure S4

Supplemental Figure S5

Supplemental Figure S6

Supplemental Figure S7

Supplemental Figure S8

Supplemental Figure S9

Supplemental Information
