# Supplementary figures and images for: PFKFB3 inhibition reprograms malignant pleural mesothelioma to nutrient stress-induced macropinocytosis and ER stress as independent binary adaptive responses
Source: Cell Death Dis. 2019 Sep 27;10(10):725. doi: 10.1038/s41419-019-1916-3 (PMC6764980; doi:10.1038/s41419-019-1916-3)

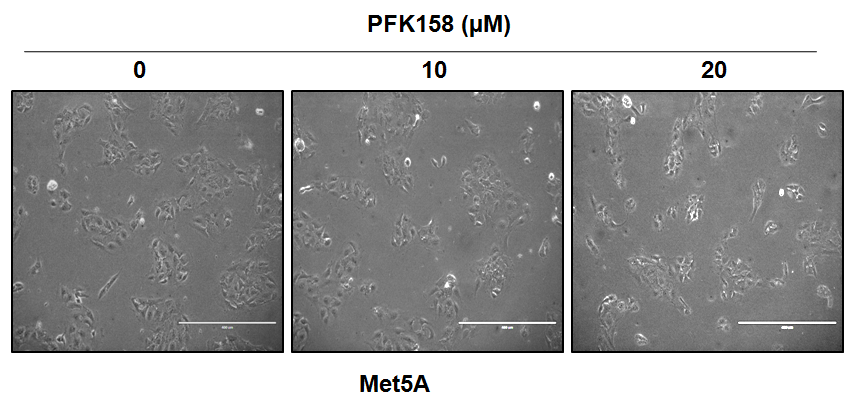

Supplement: Supplementary file 1 — Supplemental Figure S1 [file 41419_2019_1916_MOESM1_ESM.tif]

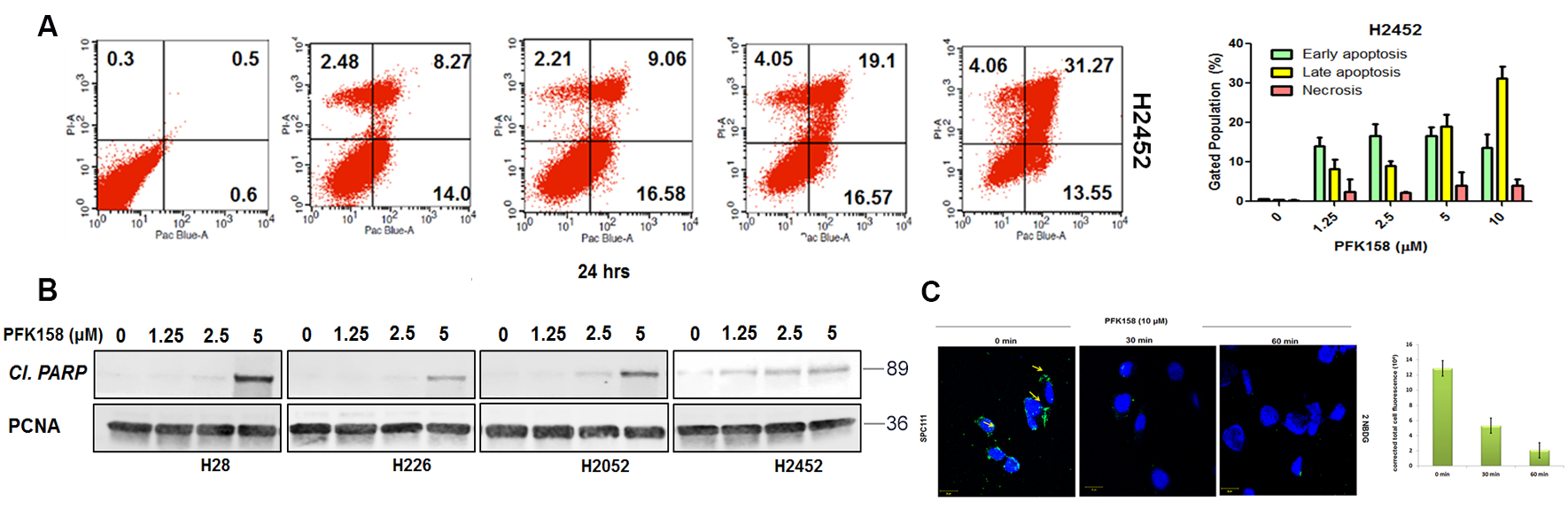

Supplement: Supplementary file 2 — Supplemental Figure S2 [file 41419_2019_1916_MOESM2_ESM.tif]

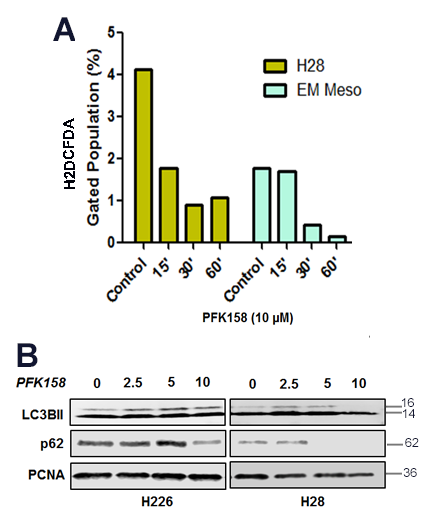

Supplement: Supplementary file 3 — Supplemental Figure S3 [file 41419_2019_1916_MOESM3_ESM.tif]

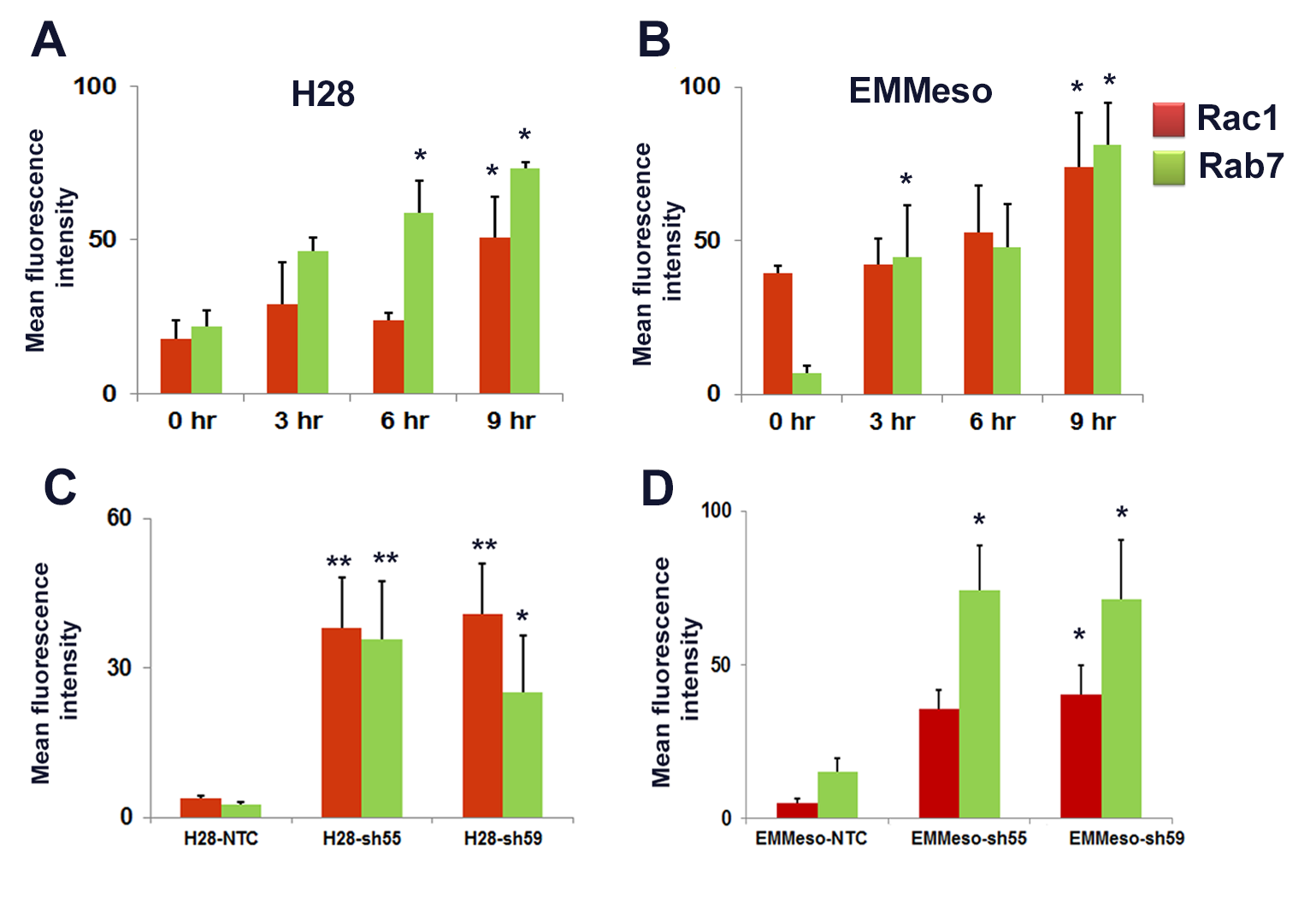

Supplement: Supplementary file 4 — Supplemental Figure S4 [file 41419_2019_1916_MOESM4_ESM.tif]

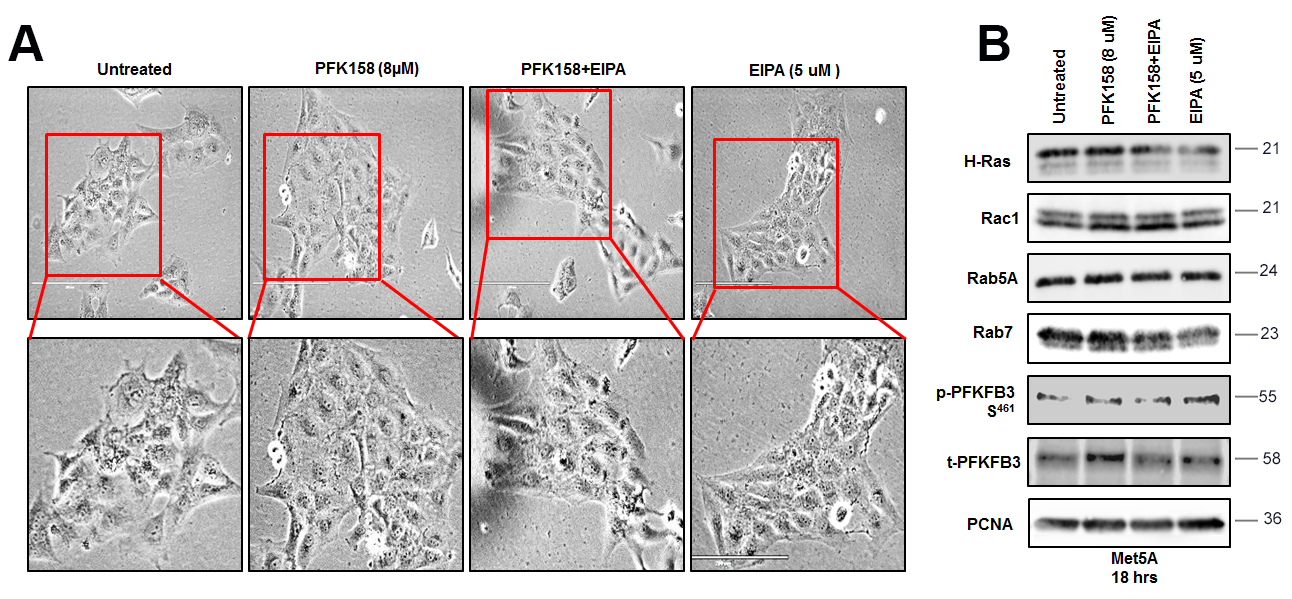

Supplement: Supplementary file 5 — Supplemental Figure S5 [file 41419_2019_1916_MOESM5_ESM.tif]

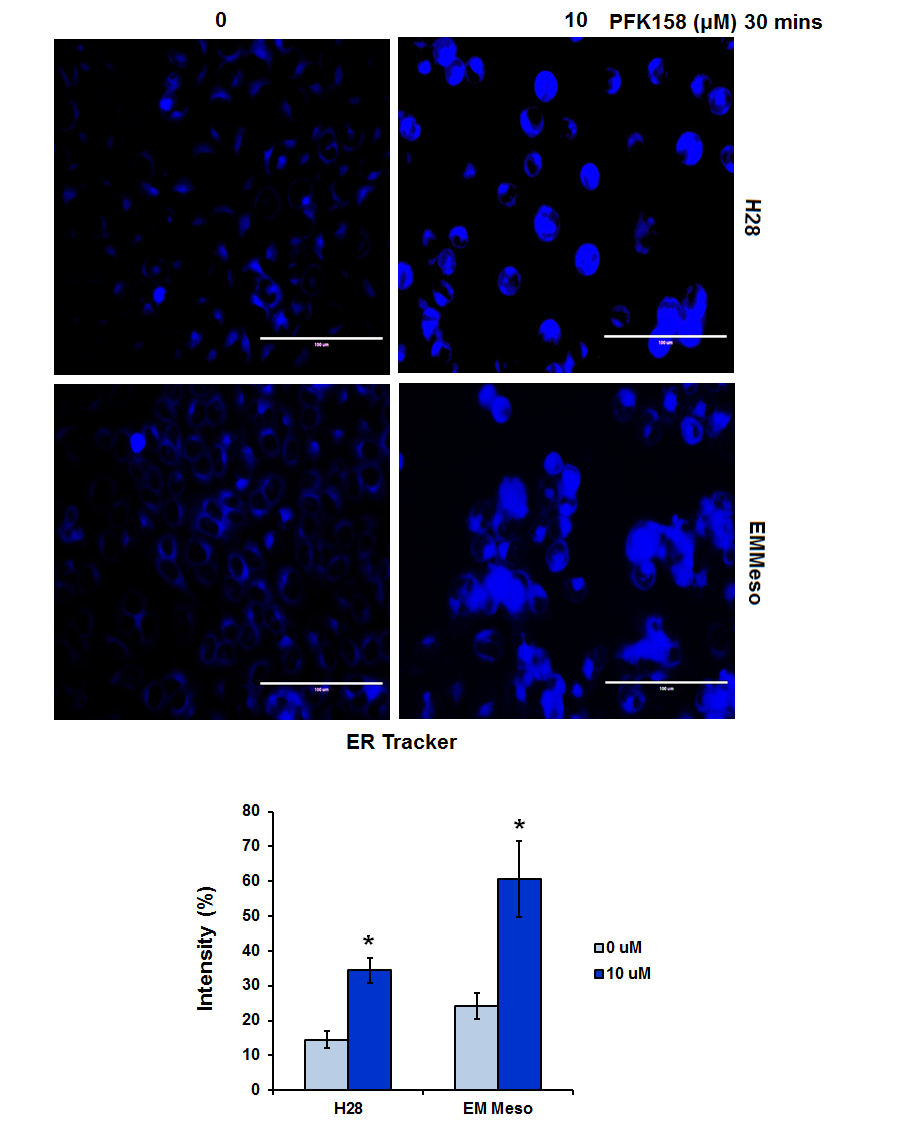

Supplement: Supplementary file 6 — Supplemental Figure S6 [file 41419_2019_1916_MOESM6_ESM.tif]

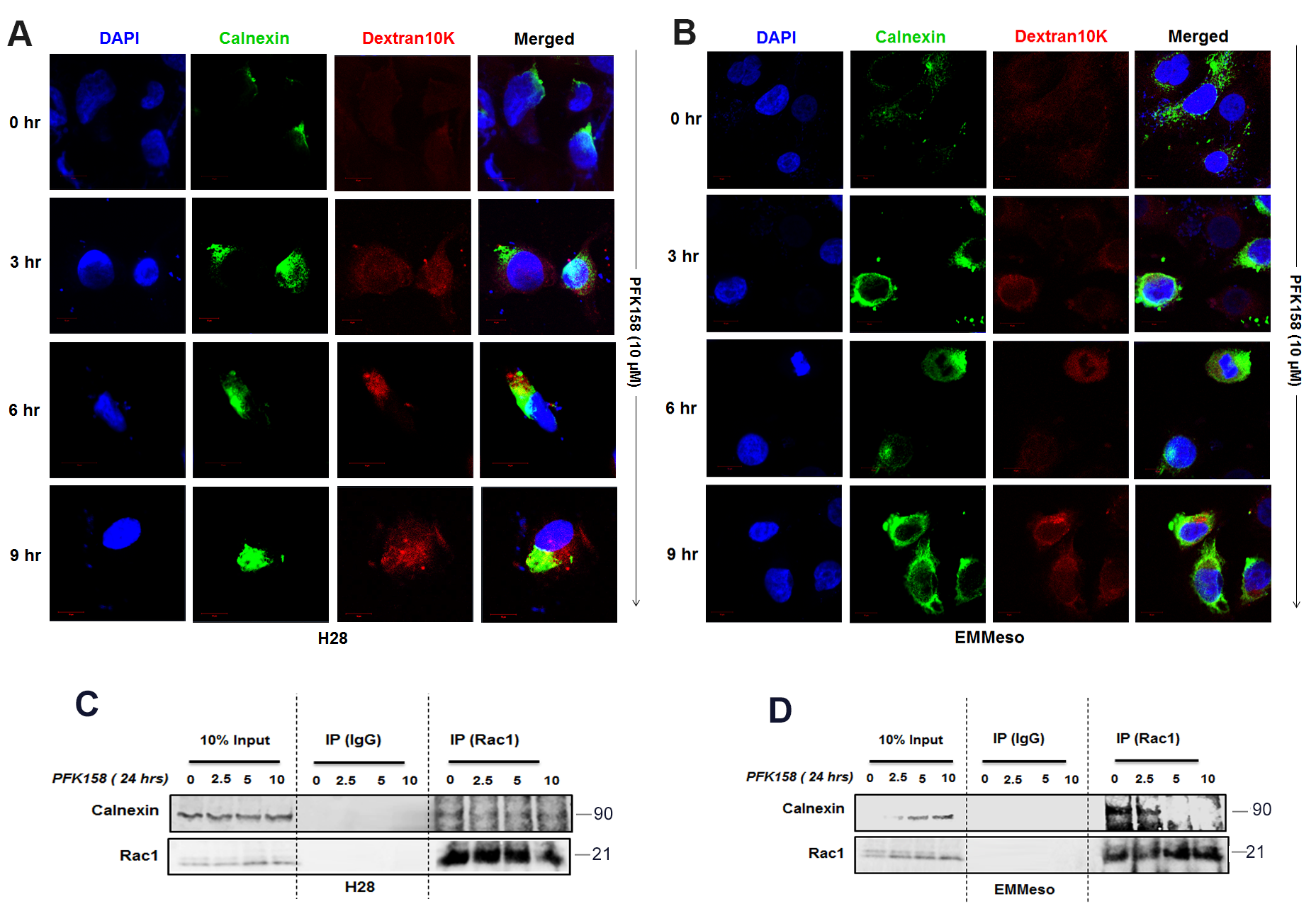

Supplement: Supplementary file 7 — Supplemental Figure S7 [file 41419_2019_1916_MOESM7_ESM.tif]

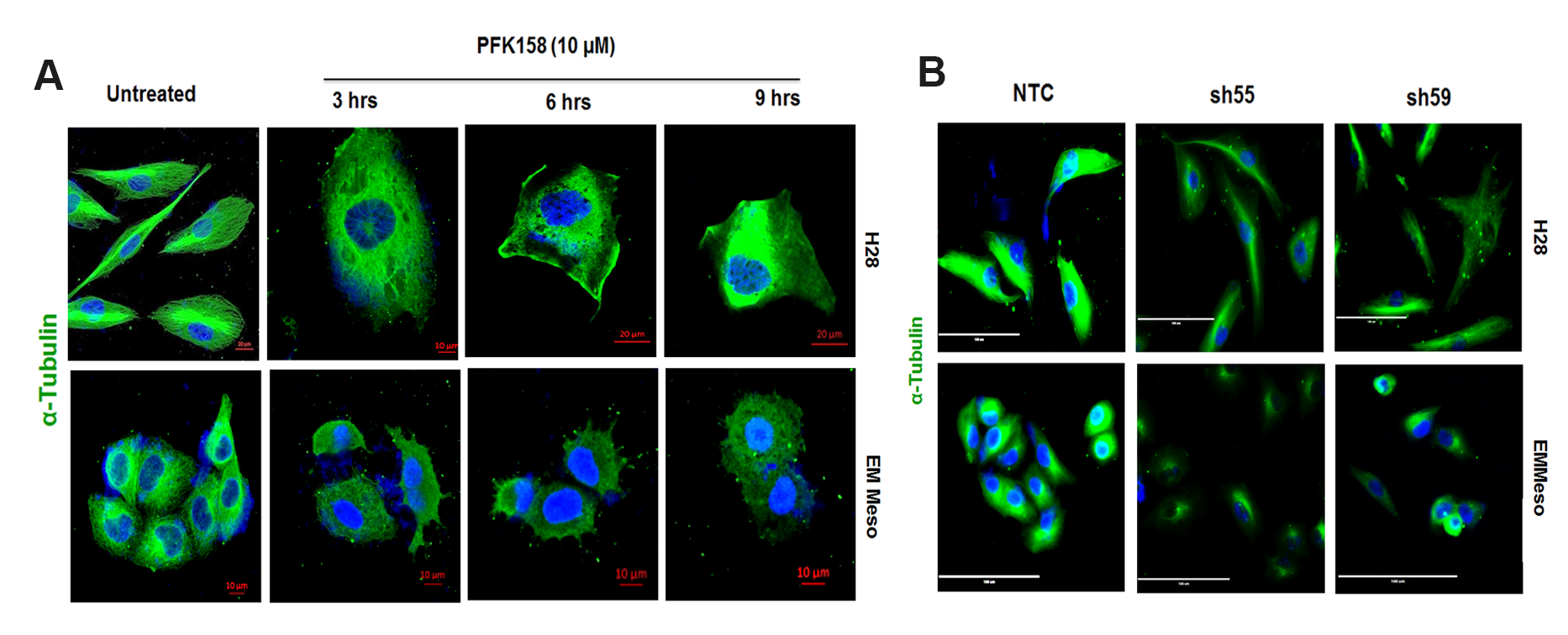

Supplement: Supplementary file 8 — Supplemental Figure S8 [file 41419_2019_1916_MOESM8_ESM.tif]

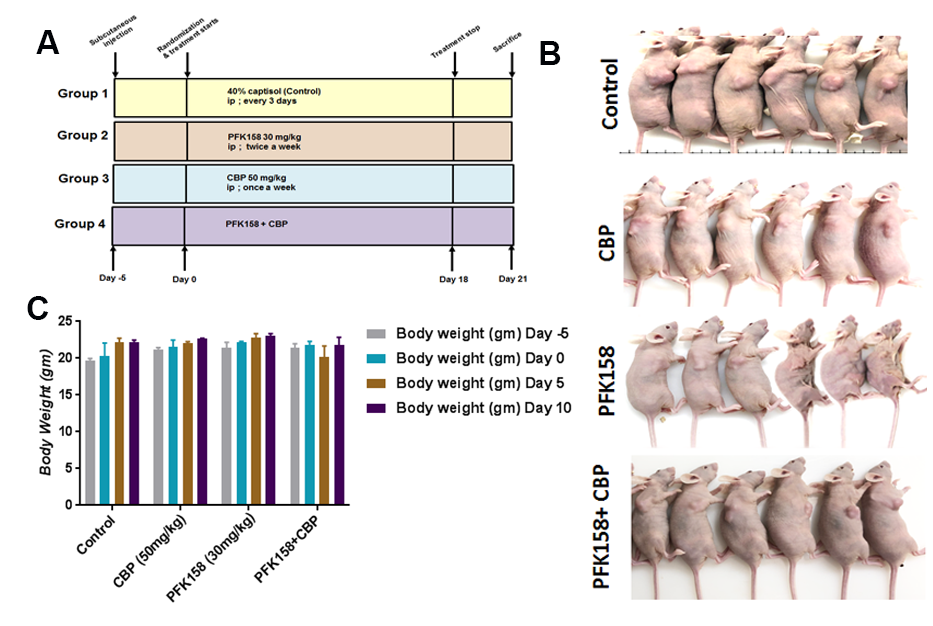

Supplement: Supplementary file 9 — Supplemental Figure S9 [file 41419_2019_1916_MOESM9_ESM.tif]
