# Supplementary material for: PFKFB3 inhibition reprograms malignant pleural mesothelioma to nutrient stress-induced macropinocytosis and ER stress as independent binary adaptive responses
Source: Cell Death Dis. 2019 Sep 27;10(10):725. doi: 10.1038/s41419-019-1916-3 (PMC6764980; doi:10.1038/s41419-019-1916-3)
Supplement: Supplementary file 10 — Supplemental Information [file 41419_2019_1916_MOESM10_ESM.docx]

| **Antibodies** | **Company** | **Catalog No.** | **Molecular Weight** |
| --- | --- | --- | --- |
| ATF4 | Cell Signaling Technology, Danvers, MA | cst11815S | 49 |
| BiP | Cell Signaling Technology, Danvers, MA | cst3177S | 78 |
| Calnexin | Santa Cruz Biotechnology, Texas, U.S.A | sc6465 | 90 |
| CHOP/GADD153 | Cell Signaling Technology, Danvers, MA | sc7351 | 30 |
| Cleaved PARP | Cell Signaling Technology, Danvers, MA | cst5625 | 89 |
| H-Ras | Santa Cruz Biotechnology, Texas, U.S.A | sc-34 | 21 |
| Lamp2 | Santa Cruz Biotechnology, Texas, U.S.A | sc-71490 | 120 |
| LC3B | Cell Signaling Technology, Danvers, MA | cst3868 | 14,16 |
| P62 | Cell Signaling Technology, Danvers, MA | cst5114S | 62 |
| PCNA | Santa Cruz Biotechnology, Texas, U.S.A | sc9857 | 36 |
| p-eIF2α(S^51^) | Abcam, Cambridge, UK | ab32157 | 36 |
| PFKFB3 | Abcam, Cambridge, UK | ab181861 | 58 |
| p-PERK(T^980^) | Cell Signaling Technology, Danvers, MA | cst3179S | 170 |
| p-PFKFB3 (S^461^) | Genescript Inc | Custom made | 55 |
| Rab5A | Millipore, USA | MABT182 | 24 |
| Rab7 | Cell Signaling Technology, Danvers, MA | cst9367T | 23 |
| Rac1 | Millipore, USA | 05-389 | 21 |
| t-eIF2α | Santa Cruz Biotechnology, Texas, U.S.A | sc133132 | 36 |
| t-PERK | Cell Signaling Technology, Danvers, MA | cst 5683S | 140 |
| Tubulin A | Santa Cruz Biotechnology, Texas, U.S.A | sc-134237 | 50 |
